# Medical Appointments in Public Hospitals

**Published:** 1888-01

**Authors:** 


### MEDICAL APPOINTMENTS IN PUBLIC HOSPITALS.

The management of public charities by politicians, and especially by the average politician of city or county governing bodies, is always attended with difficulty. Especially is this so in the selection of proper professional attendants. It therefore happens that the medical appointments are often unwise and the composition of the professional staff unstable.

For some years past, in the Cook County Hospital, the appointments have been made without much reference to the qualifications of the men appointed, even for the care of the sick, and apparently with no reference to qualification for giving clinical instruction in an institution particularly well adapted for that purpose, and affording a field of great usefulness.

During the last few months there has been a reorganization of the County Board of Commissioners, and an effort made to bring about a better state of things. The daily papers announce that each Commissioner has nominated one member for the Medical Board for the hospital, and that most of the nominees have been accepted by the Board. Some good men are on the staff. How much, if anything, others have paid for appointments is not known. It seems probable, if not certain, that the value of the hospital as a means of clinical teaching has been very much crippled in the past by the fact that men not qualified to teach have been able to buy positions on the staff. Whether the "Reform Board" has brought about reforms in this respect may well be questioned.

